# Constitutive expression of spliced X-box binding protein 1 inhibits dentin formation in mice

**DOI:** 10.3389/fphys.2023.1319954

**Published:** 2024-01-10

**Authors:** Qian Xu, Jiahe Li, Hua Zhang, Suzhen Wang, Chunlin Qin, Yongbo Lu

**Affiliations:** Department of Biomedical Sciences, Texas A&M University School of Dentistry, Dallas, TX, United States

**Keywords:** X-box binding protein 1 (XBP1), tooth development, odontoblast, cell differentiation, dentin, dentinogenesis

## Abstract

Upon endoplasmic reticulum (ER) stress, inositol-requiring enzyme 1 (IRE1) is activated, which subsequently converts an unspliced X-box binding protein 1 (*XBP1U*) mRNA to a spliced mRNA that encodes a potent XBP1S transcription factor. XBP1S is essential for relieving ER stress and secretory cell differentiation. We previously established *Twist2-Cre*;*Xbp1* ^*CS/+*^ mice that constitutively expressed XBP1S in the *Twist2*-expressing cells as well as in the cells derived from the *Twist2*-expressing cells. In this study, we analyzed the dental phenotype of *Twist2-Cre*;*Xbp1* ^*CS/+*^ mice. We first generated a mutant *Xbp1s* minigene that corresponds to the recombinant *Xbp1* ^Δ26^ allele (the *Xbp1* ^*CS*^ allele that has undergone Cre-mediated recombination) and confirmed that the *Xbp1s* minigene expressed XBP1S that does not require IRE1α activation *in vitro*. Consistently, immunohistochemistry showed that XBP1S was constitutively expressed in the odontoblasts and other dental pulp cells in *Twist2-Cre*;*Xbp1* ^*CS/+*^ mice. Plain X-ray radiography and µCT analysis revealed that constitutive expression of XBP1S altered the dental pulp chamber roof- and floor-dentin formation, resulting in a significant reduction in dentin/cementum formation in *Twist2-Cre*;*Xbp1* ^*CS/+*^ mice, compared to age-matched *Xbp1* ^*CS/+*^ control mice. However, there is no significant difference in the density of dentin/cementum between these two groups of mice. Histologically, persistent expression of XBP1S caused a morphological change in odontoblasts in *Twist2-Cre*;*Xbp1* ^*CS/+*^ mice. Nevertheless, *in situ* hybridization and immunohistochemistry analyses showed that continuous expression of XBP1S had no apparent effects on the expression of the *Dspp* and *Dmp1* genes. In conclusion, these results support that sustained production of XBP1S adversely affected odontoblast function and dentin formation.

## 1 Introduction

X-box binding protein 1 (XBP1) is a member of the family of basic leucine zipper (bZIP) transcription factors (Liou et al., 1990). *Xbp1* is highly expressed in the secretory cells including odontoblasts in tooth and osteoblasts in bone as well as in exocrine glands including pancreas and salivary glands during mouse embryonic development (Clauss et al., 1993). The *Xbp1* gene expresses an unspliced *XBP1* (*XBP1U*) mRNA that encodes a transcription factor XBP1U that is subject to rapid degradation (Yoshida et al., 2001; Tirosh et al., 2006; Yoshida et al., 2006; Navon et al., 2010; Luo et al., 2022). XBP1U contains a DNA-binding domain in its amino-terminal region but does not have a transcriptional activation domain, thereby it cannot activate transcription of a gene (Yoshida et al., 2001; Calfon et al., 2002; Tirosh et al., 2006; Navon et al., 2010; Luo et al., 2022). The primary function of XBP1U is to regulate the stability of other transcription factors at the post-translational level in a variety of biological processes (Yoshida et al., 2006; Zhao et al., 2013; Martin et al., 2014; Huang et al., 2017; Zhao et al., 2017; Yang et al., 2022).

Inositol-requiring enzyme 1α (IRE1α) is a Type I transmembrane protein in the endoplasmic reticulum (ER) that is highly conserved across species (Tirasophon et al., 1998; Wang et al., 1998). It consists of an N-terminal ER luminal domain, a transmembrane domain, and a cytosolic domain with serine/threonine kinase and endoribonuclease (RNase) activities (Cox et al., 1993; Shamu and Walter, 1996; Tirasophon et al., 1998). When an accumulation of misfolded/unfolded proteins occurs in the ER, a condition known as “ER stress”, IRE1α is oligomerized and autophosphorylated, resulting in activation of its RNase domain (Shamu and Walter, 1996; Welihinda and Kaufman, 1996; Tirasophon et al., 1998; Lee et al., 2008b; Korennykh et al., 2009; Oikawa et al., 2009; Li et al., 2010; Korennykh et al., 2011a; Korennykh et al., 2011b). The activated IRE1α RNase catalyzes an unconventional splicing of 26 nucleotides from the unspliced *XBP1U* mRNA to give rise to a spliced *XBP1* (*XBP1S*) mRNA that is translated into a highly active transcription factor XBP1S (Yoshida et al., 2001; Calfon et al., 2002; Lee et al., 2002). XBP1S shares the same N-terminal DNA-binding domain as XBP1U, but it also has a transcriptional activation domain in its carboxy-terminal region, due to the reading frameshift in *XBP1S* mRNA caused by the splicing (Yoshida et al., 2001; Calfon et al., 2002; Lee et al., 2002). XBP1S enters the nucleus and activates the transcription of the genes encoding the proteins involved in protein folding, ER-associated degradation (ERAD) and lipid biosynthesis, which together help alleviate ER stress (Lee et al., 2003; Yoshida et al., 2003; Shaffer et al., 2004; Sriburi et al., 2004; Oda et al., 2006; Acosta-Alvear et al., 2007; Lee et al., 2008a). Thereby, XBP1S plays an important role in promoting cell survival and adaptive response to ER stress.

In addition, XBP1S is essential for the normal development and function of many secretory organs/cells, such as pancreas (Lee et al., 2005), salivary glands (Lee et al., 2005), plasma cells (Reimold et al., 2000; Reimold et al., 2001; Gass et al., 2002; Iwakoshi et al., 2003; Shaffer et al., 2004), hepatocytes (Reimold et al., 2000; Lee et al., 2005) and osteoblasts (Tohmonda et al., 2011). XBP1 is required for an expansion of the ER to accommodate a high level of nascent secretory proteins as well as for the expression of the genes that code for ER chaperones to facilitate protein folding and subsequent protein trafficking through the secretory pathway during the differentiation of secretory cells (Reimold et al., 2001; Iwakoshi et al., 2003; Shaffer et al., 2004; Lee et al., 2005). Additionally, XBP1S directly promotes the transcription of the *osterix* (*Osx*) gene that encodes a transcription factor indispensable for osteoblast differentiation (Nakashima et al., 2002; Tohmonda et al., 2011). Like osteoblasts, odontoblasts are secretory cells that produce a large amount of secretory proteins, which form the organic matrix of dentin. In addition to osteoblasts, OSX is also involved in odontoblast differentiation and function (Kim et al., 2015; Zhang et al., 2015; Yang et al., 2017). However, the roles of XBP1S in odontoblast differentiation and function are largely unknown.

We previously generated a novel mouse model (referred to as “*Xbp1* ^*CS/+*^“) with a modified *Xbp1* allele that constitutively expressed XBP1S following Cre-recombinase (Cre)-mediated recombination, and showed that *Twist2-Cre*;*Xbp1* ^*CS/+*^ mice constitutively expressed XBP1S in a variety of tissues/organs (Xu et al., 2021). In this study, we examined the role of XBP1S in odontoblast differentiation and dentin formation in *Twist2-Cre*;*Xbp1* ^*CS/+*^ mice. We confirmed that XBP1S was constitutively expressed in the odontoblasts in *Twist2-Cre*;*Xbp1* ^*CS/+*^ mice. We found that persistent expression of XBP1S in mice significantly reduced dentin formation and changed odontoblast morphology, but appeared to have no obvious effects on the expression of the odontoblast differentiation markers. These findings indicate that sustained expression of XBP1S in the odontoblasts inhibited dentin formation.

## 2 Materials and methods

### 2.1 Animals

All mice were maintained on a C57BL/6 background and were bred and maintained in community housing (≤4 mice/cage, 22°C) on a 12 h light/dark cycle with free access to water and standard pelleted food. All animal procedures were approved by the Institutional Animal Care and Use Committee (IACUC) of Texas A&M University (Dallas, TX).

### 2.2 DNA constructs

**FIGURE 1:**
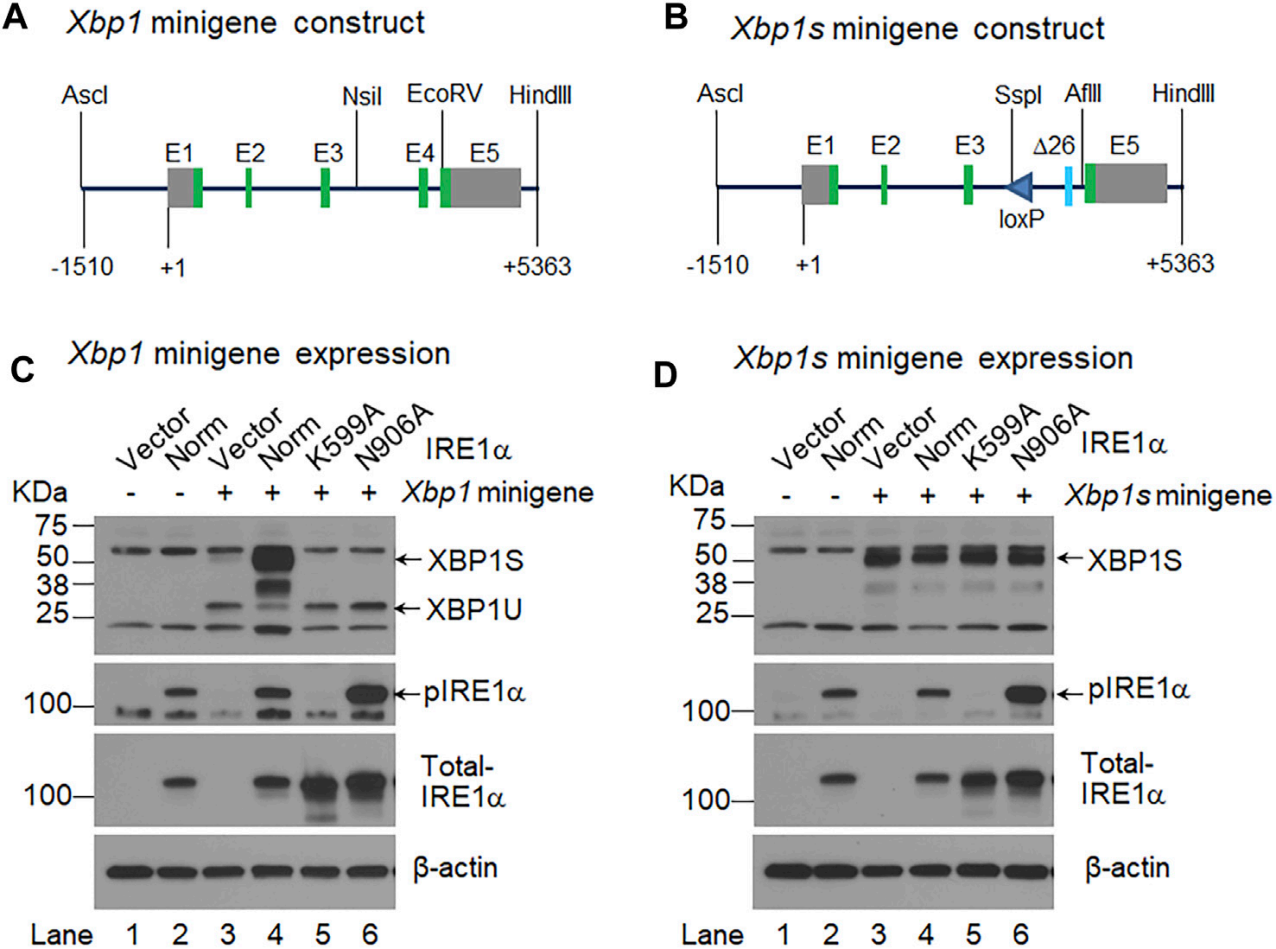
IRE1α activation-independent expression of XBP1S. **(A, B)**. Shown are the schematic representations of the *Xbp1* minigene **(A)** and *Xbp1s* minigene **(B)** constructs. The *Xbp1s* minigene contains a modified exon 4 (Δ26) that lack the 26 intronic sequence, and it also contains a loxP site in intron 3. +1, the transcription start site; E, exon; green boxes, coding exons; and grey boxes, non-coding exons. AscI, NsiI, EcoRV, HindIII, SspI and AflII are restriction endonucleases. **(C, D)**. HEK 293 cells were transiently transfected with the *Xbp1* minigene **(C)** or *Xbp1s* minigene **(D)** together with the pCDNA3 empty vector (Vector) (lane 3) or a construct expressing normal (Norm) IRE1α (lane 4) or two IRE1α variants, K599A (lane 5) and N906A (lane 6). HEK 293 cells were transfected with the pCDNA3 empty vector alone (lane 1) or the construct expressing normal IRE1α alone (lane 2) as controls. Total cell lysate was harvested 48 h after transfection and analyzed by Western-blotting with an antibody that recognizes both XBP1U and XBP1S, the blot was then stripped and sequentially probed with an antibody against phosphorylated IRE1α and total IRE1α. The blot was probed with mouse monoclonal β-actin antibody as the loading control.

Two DNA constructs, *Xbp1* minigene and *Xbp1s* minigene constructs, were generated (Figure 1). The former construct expressed an unspliced *XBP1* mRNA that could undergo an unconventional splicing to form a spliced *XBP1S* mRNA by activated IRE1α RNase, whereas the latter only produced a spliced *XBP1S* mRNA. The *Xbp1* minigene was generated from three *Xbp1* gene fragments, A, B and C. Fragment A is a 4.6-kb fragment containing promoter, exon 1, intron 1, exon 2, intron 2, exon 3 and 5′ part of intron 3. It was released from the targeting construct used to generate *Xbp1* ^*CS/+*^ mice (Xu et al., 2021) by restriction endonuclease AscI and NsiI. Fragment B is a 950-bp fragment containing 3′ part of intron 3, exon 4, intron 4, and 5′ part of exon 5. It was released by enzymes NsiI and EcoRV from the 1.3 kb PCR product amplified from the genomic DNA extracted from a wild-type C57/BL6 mouse using the following primers, Xbp1-F and Xbp1-R1, as previously described (Xu et al., 2021). Fragment C is a 1.3-kb fragment containing most of exon 5. It was first released from the targeting construct (Xu et al., 2021) by restriction enzymes EcoRV and Hind III, and subcloned into the EcoRV and HindIII sites of pBluescript SK(−) vector to generate an intermediate construct which appended a SalI cut site to the 3′ terminus of fragment C; fragment C was subsequently released from the intermediate construct by restriction enzymes EcoRV and SalI. Fragments A, B and C were then ligated into the MluI and SalI sites of a pGL3-basic vector (Promega) to replace the luciferase gene and generate the *Xbp1* minigene (Figure 1A). The *Xbp1s* minigene was also generated from three DNA fragments A′, B′ and C’. Fragment A′ is a 4.7-kb fragment containing promoter, exon 1, intron 1, exon 2, intron 2, exon 3 and 5′ part of intron 3. It was released from the targeting construct by restriction endonucleases AscI and SspI. Fragment B′ is a 700-bp fragment containing 3′ part of intron 3, a modified exon 4 lacking the 26 intronic sequence (Δ26), and 5′ part of intron 4. It was released by enzymes SspI and AflII from the 1.3 kb PCR products amplified from the recombinant *Xbp1* allele of a *Twist2-Cre*;*Xbp1* ^*CS/CS*^ mouse using the following primers, Xbp1-F and Xbp1-R1, as previously described (Xu et al., 2021). Fragment C′ is a 1.5-kb fragment containing 3′ part of intron 4 and exon 5. It was released from the *Xbp1* minigene by restriction endonucleases AflII and SalI. Fragments A′, B′ and C’ were then ligated into the MluI and SalI sites of the pGL3-basic vector to replace the luciferase gene and generate the *Xbp1s* minigene (Figure 1B). The *Xbp1s* minigene ended up with a loxP site in intron 3. All restriction endonucleases were purchased from New England Biolabs. In addition, a plasmid expressing human IRE1α was a gift from Dr. Randal Kaufman (Addgene plasmid # 21892). The plasmid encoding a kinase-defective IRE1α-K599A (Tirasophon et al., 1998) or RNase-defective IRE1α-N906A variant (Han et al., 2009) was generated using QuikChange II XL site-directed mutagenesis kit (Agilent Technologies, Texas, USA). All DNA constructs were confirmed by restriction enzyme digestion and/or and DNA sequencing.

### 2.3 Cell culture and DNA transfection

HEK293 EBNA cells were grown and maintained in Dulbecco’s modified Eagle’s medium (DMEM) (Corning, USA) supplemented with 10% heat inactivated fetal bovine serum (FBS), GlutaMAX™ and penicillin/streptomycin (Gibco, USA) at 37°C with 5% CO_2_ and 95% humidity, as described previously (Liang et al., 2019). DNA transfections were performed using X-tremeGENE™ 9 transfection reagent (Roche, Mannheim, Germany), according to the manufacturer’s instruction. Briefly, HEK293 cells were plated into a 6-well plate at a density of 8 × 10^5^ cells per well; on the next day, the cells were transiently transfected with a total of 3 µg of the *Xbp1* or *Xbp1s* minigene construct together with the pCDNA3 empty vector or a construct expressing normal IRE1α or two IRE1α variants, K599A and N906A. HEK293 cells were transfected with the empty vector alone or the construct expressing normal IRE1α together with the empty vector as controls. Total cell lysates were harvested 48 h after transfection and analyzed by Western-blotting analysis as described below.

### 2.4 Western-blotting analysis

Western-blotting analysis was performed as previously described (Liang et al., 2019). Briefly, 40 µg of the total cell lysates were electrophoresed on a 4%–15% gradient SDS-PAGE (sodium dodecyl sulfate-polyacrylamide gel electrophoresis) gel (BioRad, Hercules, CA), and the proteins were subsequently transferred onto a PVDF membrane (EMD Millipore, Billerica, MA). The membrane was sequentially immunoblotted with rabbit anti-XBP1 polyclonal antibody that recognizes both XBP1U and XBP1S (1:4000, Abcam, Cambridge, MA), horseradish peroxidase (HRP)-conjugated rabbit polyclonal anti-phosphorylated IRE1α (pSer724) (1:5000; Novus Biologicals) and mouse monoclonal anti-IRE1α antibody (1:1000; Santa Cruz Biotechnology, Inc.). The secondary antibodies used included HRP-conjugated goat anti-rabbit IgG antibody (1:2000; Santa Cruz Biotechnology, Inc.) and HRP-conjugated goat anti-mouse IgG antibody (1:2000; Santa Cruz Biotechnology), β-actin was immunoblotted with peroxidase-conjugated mouse monoclonal anti-β-actin antibody (1:50,000; Sigma). The immunostained protein bands were detected with ECL™ Chemiluminescent detection reagents (Pierce Biotechnology, Inc., Rockford, IL), and imaged by using a CL-XPosure film (Pierce Biotechnology).

### 2.5 Generation of *Twist2-cre*;*Xbp1* ^*CS/+*^ mice

The *Xbp1* ^*CS/+*^ female mice were mated with *Twist2-Cre* knock-in male mice (*Twist2* ^*Cre/+*^; Stock No. 008712, the Jackson Laboratory) to generate *Twist2-Cre*;*Xbp1* ^*CS/+*^ mice, as previously described (Xu et al., 2021). *Twist2-Cre* expresses Cre recombinase in the dental mesenchyme that later gives rise to odontoblasts (Yu et al., 2003; Meng et al., 2015). The *Xbp1* ^*CS/+*^ mice carries a modified *Xbp1* allele that constitutively express spliced XBP1S following Cre-recombinase (Cre)-mediated recombination event. Thus, XBP1S was constitutively expressed in the odontoblasts and their progenitors in *Twist2-Cre*;*Xbp1* ^*CS/+*^ mice. The dental phenotype of *Twist2-Cre*;*Xbp1* ^*CS/+*^ mice were analyzed, in comparison with age-matched *Xbp1* ^*CS/+*^ control mice. Both male and female mice were analyzed as no phenotypic differences were noted between different sexes. PCR genotyping was performed using genomic DNA extracted from mouse tail biopsies, as previously described (Xu et al., 2021).

### 2.6 Plain X-ray radiography and micro-computed tomography (μCT)

The mandibles were dissected from 3- and 7-week-old *Xbp1* ^*CS/+*^ and *Twist2-Cre*;*Xbp1* ^*CS/+*^ mice and fixed in 4% paraformaldehyde (PFA) in diethylpyrocarbonate (DEPC) - treated 0.1 M phosphate-buffered saline (PBS) overnight. The left halves of the mandibles were then stored in 70% ethanol for plain x-ray radiography and µCT analyses, and the right halves were processed for histological analysis (see below). For plain x-ray radiography analysis, the left halves of the mandibles were analyzed by a high-resolution Faxitron X-Ray MX-20 Specimen Radiography System (Faxitron X-Ray Corp., Tucson, AZ) at 6s/26 kV for 3-week-old mice and at 10.6s/26 kV for 7-week-old mice. For µCT analysis, the first molars of the left halves of the mandibles were scanned with a high-resolution Scanco μCT35 imaging system (Scanco Medical, Brüttisellen, Switzerland) in 6-μm slice increment at 70 kV, 116 μA, as previously described (Zhang et al., 2018; Chavez et al., 2021). For three-dimensional (3D) structure construction and morphometric analysis of the mandibular first molars, the whole teeth were outlined. Thresholds were determined for each age based on visual comparisons that could distinguish the tissue of interest from the surrounding tissues (Christiansen, 2016). For dentin and cementum, a threshold of 250 was used for 3-week-old mice, whereas a threshold of 270 was used for 7-week-old mice. For measuring roof and floor dentin thickness, the lowest point at the upper border of the roof dentin concave and the highest point at the lower border of the floor dentin convex were taken as reference points. The roof dentin thickness and floor dentin thickness were defined as the thickness of dentin on the line determined by the two reference points on the sagittal plane that transverses the center of the mandibular first molars. The center of the mandibular first molar was defined as the sagittal (mesial to distal) section crossing both the most proximal and distal pulp horns, which usually bring two more pulp horns between them, and with the largest openings of both proximal and distal root apexes. The central 10 slices were measured for the roof dentin thickness and floor dentin thickness for each mouse. An average of 10 measurements were taken as the thickness of roof dentin and floor dentin, respectively, for each mouse. The morphometric parameters, including the volume and density, were evaluated using the μCT built-in software. The data obtained from 3 to 5 independent mice for each genotype were used for quantitative analysis.

### 2.7 Histological analysis

The right halves of the mandibles from the 3- and 7-week-old *Xbp1* ^*CS/+*^ and *Twist2-Cre*;*Xbp1* ^*CS/+*^ mice were used for histological analysis. Following fixation, the mandibles were decalcified in 15% ethylenediaminetetraacetic acid (EDTA) solution (pH 7.4) at 4°C for 7 days to 2 weeks, depending on the age of the mice. The decalcified mandibles were then dehydrated in a series of gradient ethanol (50% ethanol for 1 h, 70% for 1 h, 95% for 2 h and 100% for 1 h twice and 100% overnight), followed by incubation in xylene for 1 h twice. The mandibles were subsequently embedded in paraffin, and were cut into serial mesio-distal sections at a thickness of 5 μm for Hematoxylin and Eosin (H&E) staining and other histological analyses. Images were taken using Leica DM4 B upright microscope equipped with a Flexacam C1 camera (Leica Biosystems, Wetzlar, Germany).

### 2.8 *In situ* hybridization

*In situ* hybridization (ISH) was performed as previously described (Gibson et al., 2013; Liang et al., 2019). Briefly, the 5-µm tissue sections were processed in xylene and gradient ethanol for dewax and rehydration, followed by antigen retrieval with 10 μg/mL protease K (Ambion, Austin, TX) for 5 min at room temperature. The sections were then hybridized with 1 μg/mL antisense complementary RNA (cRNA) probe at 65 °C for 14–16 h. The probes used include 1.1 kb digoxigenin (DIG)-labeled DSPP cRNA probe and 0.8 kb DIG-labeled DMP1 cRNA probe. The sections were blocked and immunostained with an anti-DIG antibody conjugated to alkaline phosphatase (1:2000, Roche, Mannheim, Germany). The signals were developed with an NBT/BCIP (nitro blue tetrazolium/5-bromo-4-chloro-3-indolyl-phosphate) chromogenic substrate system (Roche ). The sections were counterstained with nuclear fast red (Sigma, Saint Louis, MO) and mounted with Permount mounting medium (Fisher Scientific, Waltham, MA). Images were taken using Leica DM4 B upright microscope (Leica Biosystems).

### 2.9 Immunohistochemistry

Immunohistochemistry (IHC) was performed to detect total XBP1 (XBP1U and XBP1S), spliced XBP1S, DSPP and DMP1, as previously described (Gibson et al., 2013; Liang et al., 2021; Xu et al., 2021). The 5-µm tissue sections were processed in xylene and gradient ethanol for dewax and rehydration, and were then incubated in sodium citrate buffer (pH 6.0) for antigen retrieval and 3% hydrogen peroxidase (H_2_O_2_) in PBS to quench endogenous peroxidase. The sections were blocked with 3% bovine serum albumin (BSA) and 10% normal goat serum (NGS) in 0.1 M PBST (0.1M PBS with 0.1% Tween-20), and incubated with primary then secondary antibodies diluted in 2% NGS. The primary antibodies used include rabbit anti-XBP1 polyclonal antibody that recognizes both XBP1U and XBP1S (1:200, Abcam, Cambridge, MA), rabbit anti-XBP1S monoclonal antibody (E9V3E) that specifically recognizes XBP1S (1:50, Cell Signaling Technology, Danvers, MA), rabbit anti-DSPP polyclonal antibody (1:1000) (Gibson et al., 2013; Liang et al., 2019), and rabbit anti-DMP1 polyclonal antibody (1:600, #857-3) (Gibson et al., 2013). The secondary antibody used was the biotinylated goat anti-rabbit IgG (H + L) antibody (1:200, Vector Laboratories, Burlingame, CA). The immunostaining signals were visualized using DAB (3.3′-diaminobenzidine) kit (Vector Laboratories, Burlingame, CA), according to the manufacturer’s instructions. The sections were counterstained with methyl green (Sigma, Saint Louis, MO) for better visualization of tissue morphology. The sections were then mounted with Permount mounting medium (Fisher Scientific, Waltham, MA). Pictures were taken using Leica DM4 B upright microscope (Leica Biosystems).

### 2.10 Statistical analysis

Statistical analysis was conducted using the GraphPad Prism 9.0 software package (GraphPad Software, San Diego, CA). Student’s *t* test was employed to compare the difference between *Xbp1* ^*CS/+*^ and *Twist2-Cre*;*Xbp1* ^*CS/+*^ mice. The quantified results were expressed as mean ± standard deviation (SD). *p* < 0.05 was considered statistically significant.

## 3 Results

### 3.1 The *Xbp1s* minigene constitutively expressed spliced XBP1S only

We previously demonstrated that *Twist2-Cre*;*Xbp1* ^*CS/+*^ mice (bearing a recombinant *Xbp1* ^Δ26^ allele, i.e., the *Xbp1* ^*CS*^ allele that has undergone Cre-recombinase mediated recombination) constitutively expressed XBP1S (Xu et al., 2021). Here we generated two DNA constructs, *Xbp1* minigene and *Xbp1s* minigene. The *Xbp1* minigene corresponds to the wild-type *Xbp1* gene (Figure 1A), whereas the *Xbp1s* minigene carries a modified version of exon 4 (E4^Δ26^, encoding XBP1S) and a loxP site in intron 3 (Figure 1B), which is equivalent to the recombinant *Xbp1* ^Δ26^ allele in *Twist2-Cre*;*Xbp1* ^*CS/+*^ mice (Xu et al., 2021). We then co-transfected the *Xbp1* or *Xbp1s* minigene construct along with the pCDNA3 empty vector or a construct expressing normal IRE1α or two IRE1α variants, K599A and N906A into HEK293 cells, and analyzed XBP1U and XBP1S by Western-blotting analyses. The IRE1α K599A variant has a defective kinase domain (Tirasophon et al., 1998), whereas the IRE1α N906A variant retains kinase activity but is defective in RNase activity (Han et al., 2009). As expected, we found that the *Xbp1* minigene produced a high level of unspliced XBP1U but a relatively low level of spliced XBP1S. We also found that co-transfection of the construct expressing normal IRE1α, but not the construct expressing either of the two IRE1α variants, dramatically increased the level of spliced XBP1S produced by the *Xbp1* minigene (Figure 1C). In contrast, the *Xbp1s* minigene only generated spliced XBP1S, and the level of XBP1S was not altered by the presence of either normal IRE1α or its two variants (Figure 1D). Moreover, consistent with previous studies showing that IRE1α overexpression alone leads to the autophosphorylation and activation of its RNase domain (Shamu and Walter, 1996; Han et al., 2009), we found that overexpression of normal IRE1α as well as the RNase-defective N906A variant, but not the Kinase-defective K599A variant, led to the phosphorylation of IRE1α (Figures 1C, D). It is also of note that no XBP1 protein was detected in the cells transfected with either the empty vector or the construct expressing normal IRE1α alone (Figures 1C, D). These *in vitro* results further confirmed that the mutant *Xbp1* ^Δ26^ allele would constitutively express spliced XBP1S mRNA and protein, regardless of whether or not IRE1α was activated in *Twist2-Cre*;*Xbp1* ^*CS/+*^ mice.

### 3.2 Constitutive expression of XBP1S in the odontoblasts in *Twist2-Cre*;*Xbp1* ^*CS/+*^ mice

**FIGURE 2:**
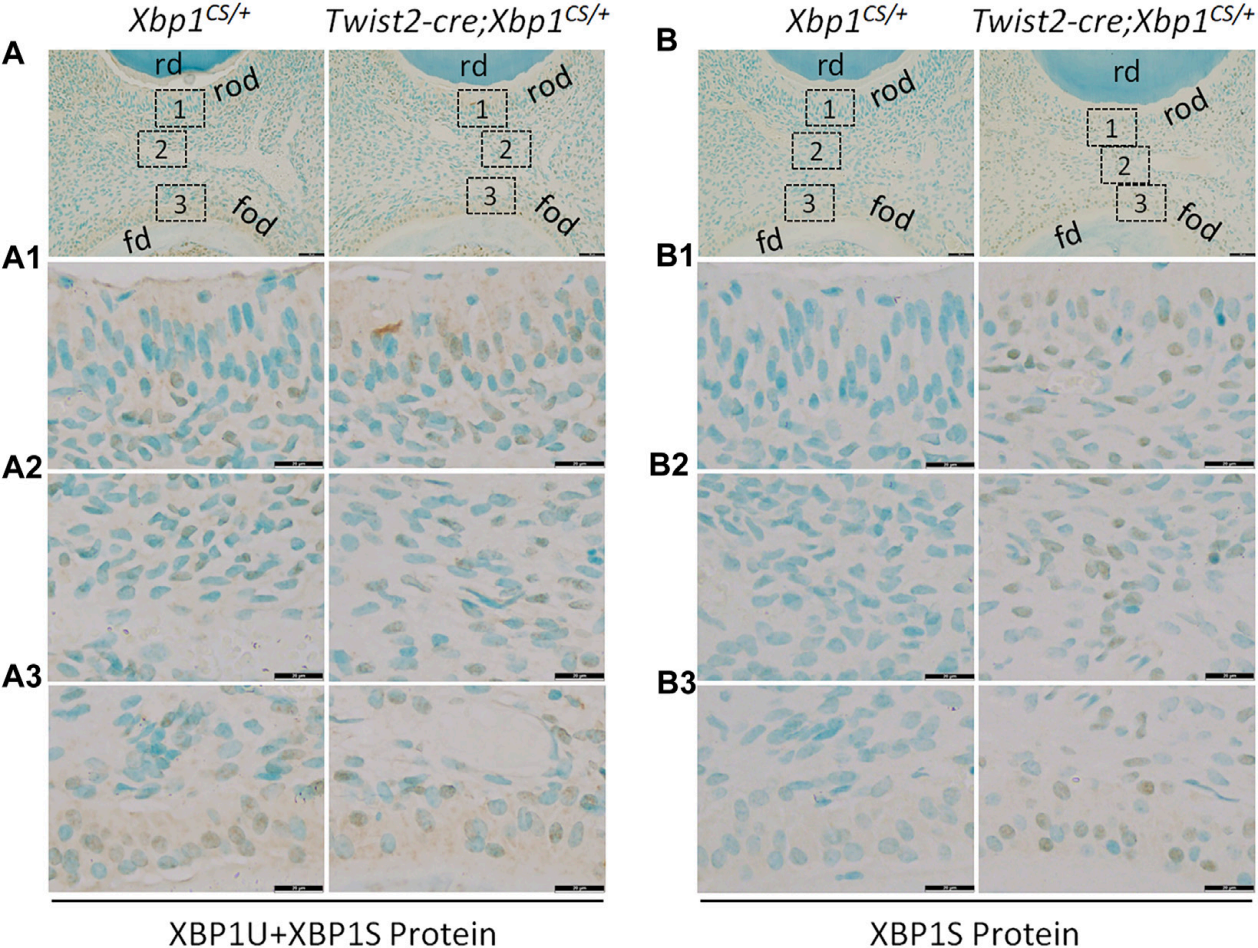
Immunohistochemical staining of total XBP1 (XBP1U and XBP1S) and XBP1S. Shown are the representative images of IHC staining of total XBP1 (including XBP1U and XBP1S) **(A)**; signal in brown) and XBP1S **(B)**; signal in brown) in the mandibular first molars of 3-week-old *Xbp1* ^*CS/+*^ and *Twist2-Cre;Xbp1* ^*CS/+*^ mice. Each image in **(A, B)** is from the middle region of the crown of a sagittally-sectioned mandibular first molar. **(A1-A3, B1-B3)** are the higher magnification views of the roof-forming odontoblasts (box1), central dental pulp cells (box 2) and floor-forming odontoblasts (box 3) in **(A, B)**, respectively. rd, roof dentin; fd, floor dentin; rod, roof-forming odontoblasts; fod, floor-forming odontoblasts. Note that the signals for total XBP1 were found in the dental pulps of *Xbp1* ^*CS/+*^ and *Twist2-Cre;Xbp1* ^*CS/+*^ mice, whereas the signals for XBP1S were strongly detected in the dental pulps of *Twist2-Cre;Xbp1* ^*CS/+*^ mice, but were barely detectable in those of *Xbp1* ^*CS/+*^ mice. Scale bars: 50 μm in **(A, B)**; 20 μm in **(A1-A3, B1-B3)**.

We next performed IHC to analyze the protein levels of XBP1U and XBP1S in the dental pulps of the mandibular first molars of 3-week-old *Xbp1* ^*CS/+*^ and *Twist2-Cre*;*Xbp1* ^*CS/+*^ mice. When an antibody that recognizes both XBP1U and XBP1S was used, we found that immunostaining signals for total XBP1 (XBP1U and XBP1S) were detected in the odontoblasts and other dental pulp cells in both *Xbp1* ^*CS/+*^ and *Twist2-Cre*;*Xbp1* ^*CS/+*^ mice (Figure 2A, A1–A3). Further, the intensity of total XBP1 immunostaining signals appeared to be comparable between these two groups of mice. However, when a monoclonal antibody that specifically recognizes XBP1S, but not XBP1U, was used, the immunostaining signals for XBP1S were observed in the odontoblasts and other dental pulp cells in *Twist2-Cre*;*Xbp1* ^*CS/+*^ mice, but were barely detectable in *Xbp1* ^*CS/+*^ mice (Figure 2B, B1, B2–B3). These results corroborated that XBP1S was constitutively expressed in the odontoblasts and other dental pulp cells in *Twist2-Cre*;*Xbp1* ^*CS/+*^ mice.

### 3.3 Dental defects associated with constitutive expression of XBP1S in the odontoblasts

**FIGURE 3:**
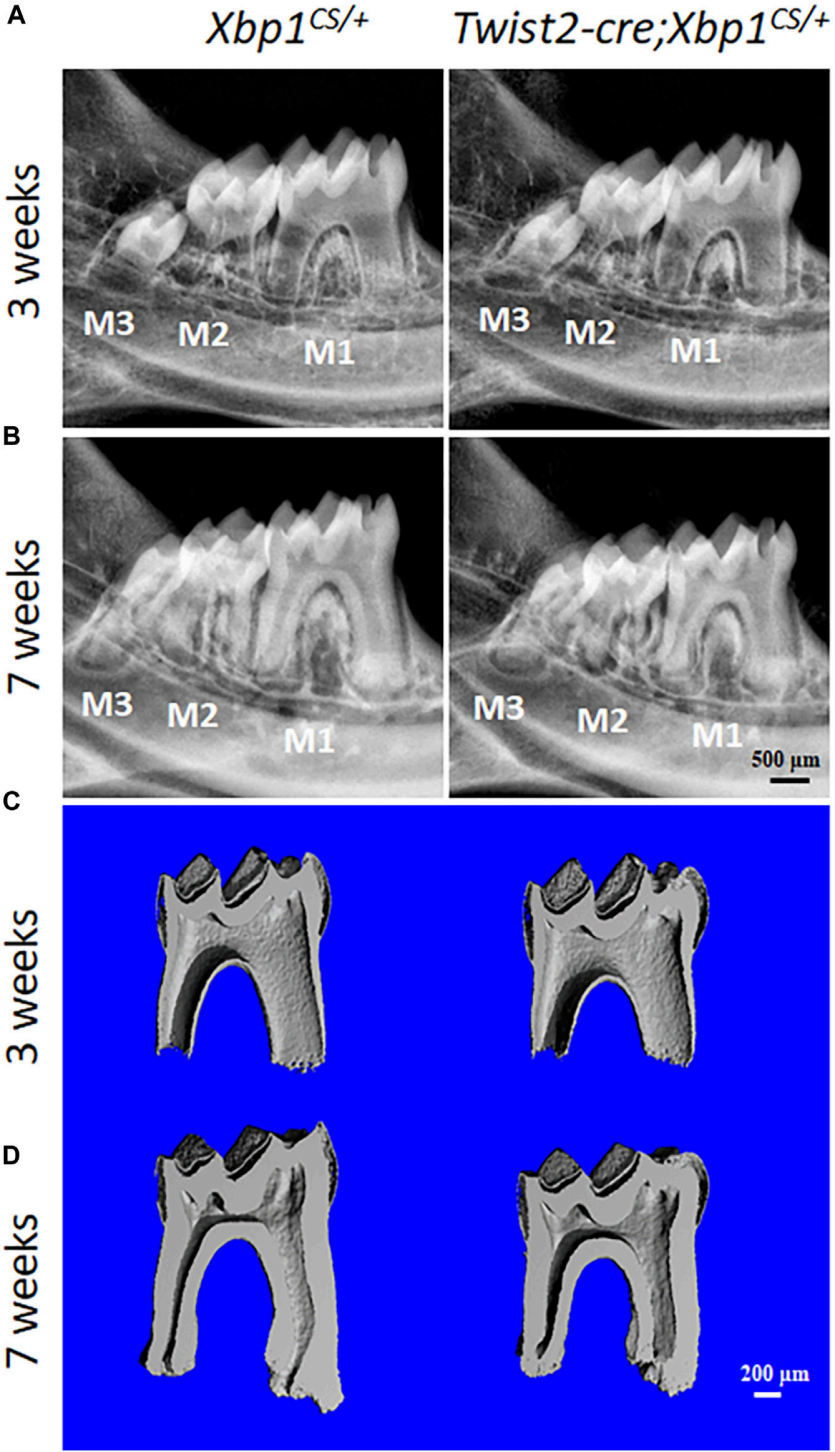
**P**lain X-ray radiography and micro-computed tomography (µCT) analyses of the mandibular molars. **(A, B)**. Representative plain X-ray radiographs of the mandibular molars of 3-week-old and 7-week-old *Xbp1* ^*CS/+*^ and *Twist2-Cre;Xbp1* ^*CS/+*^ mice. M1, first molar; M2, second molar; M3, third molar. Scale bar: 500 μm. **(C, D)**. Representative 3-dimensional reconstructed μCT images (sagittal sections) of the mandibular first molars of 3-week-old and 7-week-old *Xbp1* ^*CS/+*^ and *Twist2-Cre;Xbp1* ^*CS/+*^ mice. The mesial side of each molar is on the right, and the distal side is on the left. Scale bar: 200 μm.

**FIGURE 4:**
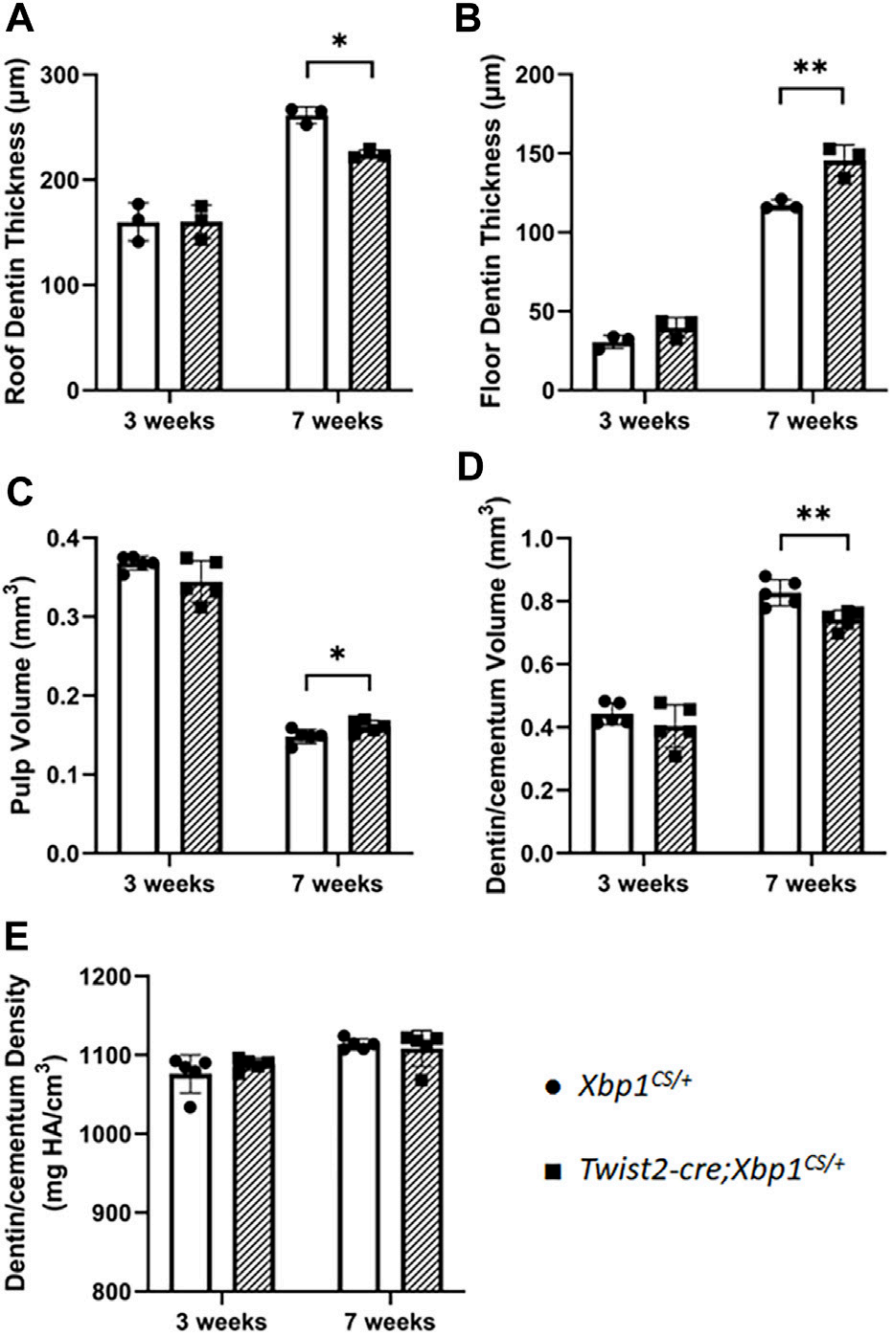
Quantitative µCT analysis of mandibular first molars. µCT analysis was performed to quantify the roof dentin thickness **(A)**, floor dentin thickness **(B)**, pulp volume **(C)**, dentin/cementum volume **(D)** and dentin/cementum density **(E)** of mandibular first molars of 3- and 7-week-old mice. Student’s t test was used to compare the difference between *Xbp1* ^*CS/+*^ and *Twist2-Cre;Xbp1* ^*CS/+*^ mice. All values are mean ± SD. n = 3 for each group in A-B; n = 5 for each group in C-E; * *p* < 0.05; ** *p* < 0.01.

We then characterized the dental phenotype of 3- and 7-week-old *Twist2-Cre*;*Xbp1* ^*CS/+*^ mice, in comparison with age-matched *Xbp1* ^*CS/+*^ control mice, by plain X-ray radiography and μCT analysis. X-ray radiography and 3D reconstructed μCT images showed that *Twist2-Cre*;*Xbp1* ^*CS/+*^ mice developed a thinner pulp chamber roof dentin and a thicker pulp chamber floor dentin by the age of 7 weeks, compared to the age-matched *Xbp1* ^*CS/+*^ mice (Figures 3A–D). Moreover, the μCT images also showed that *Twist2-Cre*;*Xbp1* ^*CS/+*^ mice form less cementum than *Xbp1* ^*CS/+*^ mice by the age of 7 weeks (Figure 3D), which was further confirmed by H&E staining (Supplementary Figure S1). Consistently, quantitative μCT analyses demonstrated that *Twist2-Cre*;*Xbp1* ^*CS/+*^ mice, by the age of 7 weeks, acquired a significant increase in the pulp floor dentin thickness and pulp volume, but a significant decrease in the pulp roof dentin thickness as well as a significant decrease in total dentin/cementum volume, compared to age-matched *Xbp1* ^*CS/+*^ control mice (Figures 4A–D). There is no significant difference in the density of dentin/cementum between these two groups of mice at either age (Figure 4E). These findings indicate that constitutive expression of XBP1S resulted in altered roof and floor dentin formation in *Twist2-Cre*;*Xbp1* ^*CS/+*^ mice.

### 3.4 Changes in odontoblast morphology in *Twist2-Cre*;*Xbp1* ^*CS/+*^ mice

**FIGURE 5:**
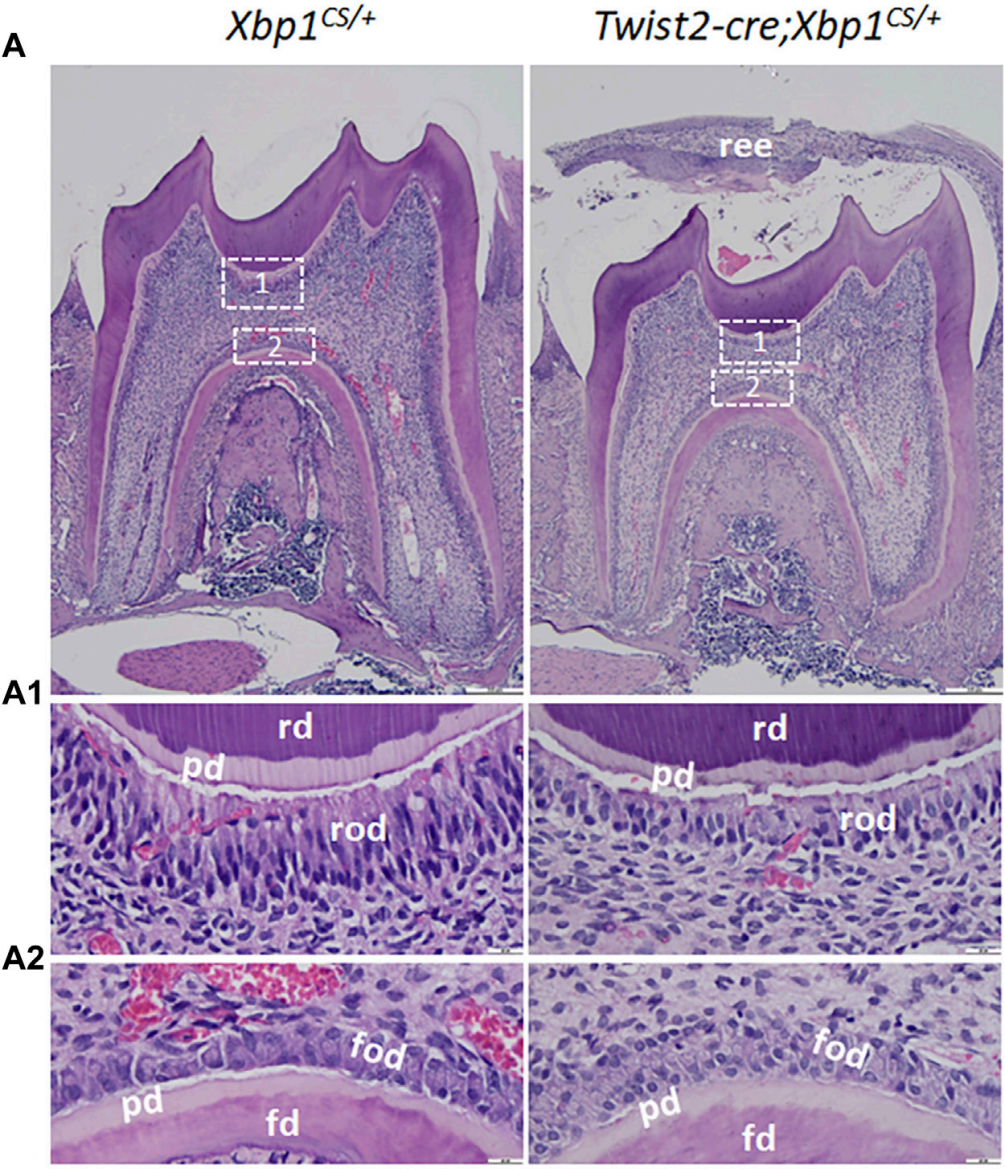
H&E staining of the mandibular first molars. **(A)**. Shown are the representative images of H&E staining of a sagittally-sectioned mandibular first molars of 3-week-old *Xbp1* ^*CS/+*^ and *Twist2-Cre;Xbp1* ^*CS/+*^ mice. **(A1, A2)** are the higher magnification views of the roof-forming odontoblasts (box1) and floor-forming odontoblasts (box 2) in A. Note that the roof-forming odontoblasts were long, columnar-shaped and highly polarized in *Xbp1* ^*CS/+*^ mice, but the roof-forming odontoblasts became shorter and irregular in *Twist2-Cre;Xbp1* ^*CS/+*^ mice. ree, reduced enamel epithelium; pd, predentin, rd, roof dentin; fd, floor dentin; rod, roof-forming odontoblasts; fod, floor-forming odontoblasts. Scale bars: 200 μm in A; 20 μm in **(A1, A2)**.

Histologically, Hematoxylin and Eosin (H&E) staining showed that the mandibular first molars of *Xbp1* ^*CS/+*^ mice had completely erupted by the age of 3 weeks, whereas those in age-matched *Twist2-Cre*;*Xbp1* ^*CS/+*^ mice were still covered by reduced enamel epithelium, indicating delayed eruption of the mandibular first molars (Figure 5A). Moreover, the roof-forming odontoblasts in the mandibular first molars of the 3-week-old *Xbp1* ^*CS/+*^ mice were aligned as a single layer of tall columnar and highly polarized cells (Figures 5A,A1). However, the roof-forming odontoblasts in *Twist2-Cre*;*Xbp1* ^*CS/+*^ mice were dramatically shorter and irregular (Figures 5A,A1). The morphological differences were less apparent in the floor-forming odontoblasts between these two groups of mice (Figures 5A,A2). Moreover, the predentin of the pulp chamber roof in *Twist2-Cre*;*Xbp1* ^*CS/+*^ mice was thinner than that of *Xbp1* ^*CS/+*^ mice (Figure 5A1). These results demonstrate that persistent expression of XBP1S altered odontoblast morphology in *Twist2-Cre*;*Xbp1* ^*CS/+*^ mice.

### 3.5 Odontoblast differentiation in *Twist2-Cre*;*Xbp1* ^*CS/+*^ mice

**FIGURE 6:**
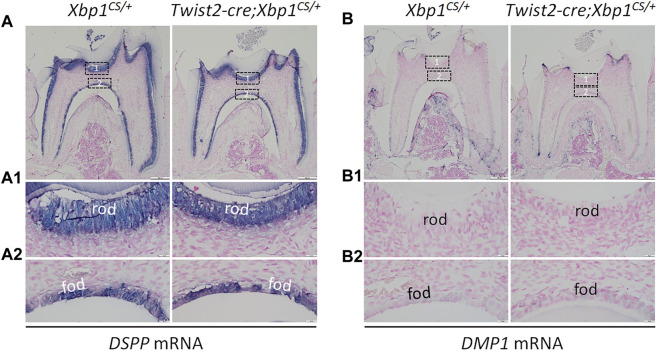
*In situ* hybridization analyses of *DSPP* and *DMP1* mRNA. Shown are the representative *in situ* hybridization analyses of *DSPP* mRNA **(A)**; signal in purple) and *DMP1* mRNA **(B)**; signal in purple) in the mandibular first molars of 3-week-old *Xbp1* ^*CS/+*^ and *Twist2-Cre;Xbp1* ^*CS/+*^ mice. Each image in **(A, B)** is from the middle region of the crown of a sagittally-sectioned mandibular first molar. **(A1-A2, B1-B2)** are the higher magnification views of the roof-forming odontoblasts (box1) and floor-forming odontoblasts (box 2) in **(A, B)**, respectively. rod, roof-forming odontoblasts; fod, floor-forming odontoblasts. Scale bars: 200 μm in (**A, B)**; 20 μm in **(A1-A2, B1-B2)**.

**FIGURE 7:**
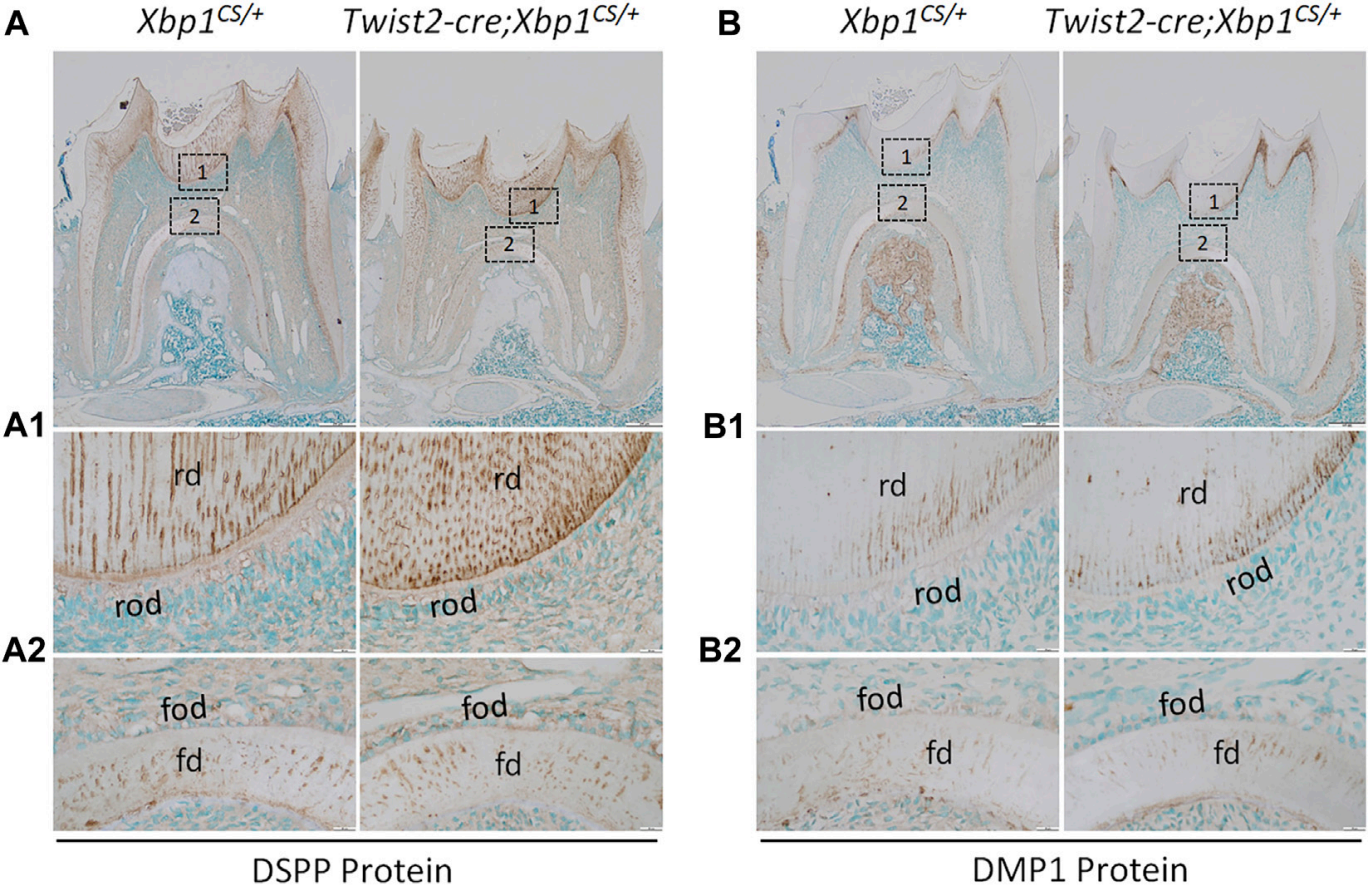
Immunohistochemical staining of DSP/DSPP and DMP1 protein. Shown are the representative images of IHC staining of DSP/DSPP **(A)**; signal in brown) and DMP1 **(B)**; signal in brown) in the mandibular first molars of 3-week-old *Xbp1* ^*CS/+*^ and *Twist2-Cre;Xbp1* ^*CS/+*^ mice. Each image in **(A, B)** is from the middle region of the crown of a sagittally-sectioned mandibular first molar. **(A1-A2, B1-B2)** are the higher magnification views of box 1 and box 2 in **(A, B)**, respectively. rd, roof dentin; fd, floor dentin; rod, roof-forming odontoblasts; fod, floor-forming odontoblasts. Scale bars: 200 μm in **(A, B)**; 20 μm in **(A1-A2, B1-B2)**.

We further analyzed the expression of the odontoblast differentiation markers, including *Dspp* and *Dmp1*, in *Twist2-Cre*;*Xbp1* ^*CS/+*^ mice by *in situ* hybridization and immunohistochemistry. *In situ* hybridization showed that *DSPP* mRNAs were highly expressed in both roof- and floor-forming odontoblasts in *Xbp1* ^*CS/+*^ mice and the level of *DSPP* mRNAs in the odontoblasts in *Twist2-Cre*;*Xbp1* ^*CS/+*^ mice was comparable to that of *Xbp1* ^*CS/+*^ mice (Figure 6A, A1, A2). Immunohistochemistry demonstrated that DSP/DSPP immunostaining signals were strongly detected in the roof dentin matrix, with a relatively low level in the floor dentin matrix; and the levels of DSP/DSPP immunostaining signals in *Twist2-Cre*;*Xbp1* ^*CS/+*^ mice were similar to those in *Xbp1* ^*CS/+*^ mice (Figure 7A, A1, A2). *In situ* hybridization also showed that a low level of *DMP1* mRNAs was expressed in both roof- and floor-forming odontoblasts in *Xbp1* ^*CS/+*^ mice; and the level of *DMP1* mRNAs in *Twist2-Cre*;*Xbp1* ^*CS/+*^ mice was similar to that in *Xbp1* ^*CS/+*^ mice (Figure 6B, B1, B2). Consistently, immunohistochemistry indicated that the intensity of DMP1 immunostaining signals were comparable in the roof and floor dentin matrices in *Xbp1* ^*CS/+*^ mice and *Twist2-Cre*;*Xbp1* ^*CS/+*^ mice (Figure 7B, B1, B2). These results suggest that sustained expression of XBP1S had no obvious effects on the expression of *Dspp* and *Dmp1* in the odontoblasts in *Twist2-Cre*;*Xbp1* ^*CS/+*^ mice.

## 4 Discussion

XBP1S plays important roles in both ER stress and cell differentiation. Here we examined the roles of XBP1S in odontoblast differentiation during mouse tooth development by gain of function approach. We found that persistent expression of XBP1S in mice led to a significant reduction in dentin formation as well as a morphological change in odontoblasts, but had no apparent effects on the expression of the odontoblast differentiation markers.

Previous studies have demonstrated that XBP1S production depends on an unconventional splicing of unspliced *XBP1U* mRNA by activated IRE1α RNase (Yoshida et al., 2001; Calfon et al., 2002; Lee et al., 2002). We have shown that *Twist2-Cre*;*Xbp1* ^*CS/+*^ mice expressed a high level of XBP1S following Cre-mediated recombination by immunohistochemistry (Xu et al., 2021). To further confirm that XBP1S production was independent of IRE1α activation in these mice, we generated a wild-type *Xbp1* minigene and a mutant *Xbp1s* minigene, which corresponds to the wild-type *Xbp1* and recombinant *Xbp1* ^Δ26^ allele, respectively. As expected, we found that the *Xbp1* minigene expressed XBP1S in a way that primarily depends on IRE1α activation, when transfected into cells *in vitro*. In contrast, the *Xbp1s* minigene constitutively expressed XBP1S, regardless of whether IRE1α was activated or not. Consistent with the *in vitro* results, immunohistochemistry demonstrated that both XBP1U and XBP1S were detected in the odontoblasts and other dental pulp cells in *Xbp1* ^*CS/+*^ and *Twist2-Cre*;*Xbp1* ^*CS/+*^ mice, when an antibody that recognizes both XBP1U and XBP1S was used. However, when an antibody that only reacts with XBP1S was used, XBP1S immunostaining signals were readily found in *Twist2-Cre*;*Xbp1* ^*CS/+*^ mice, yet were barely detectable in *Xbp1* ^*CS/+*^ mice. These results confirmed that XBP1S was constitutively produced in the odontoblasts and other pulp cells in *Twist2-Cre*;*Xbp1* ^*CS/+*^ mice.

Constant production of XBP1S affected dentin formation in *Twist2-Cre*;*Xbp1* ^*CS/+*^ mice. Plain X-ray radiography and μCT analysis demonstrated that *Twist2-Cre*;*Xbp1* ^*CS/+*^ mice had enlarged dental pulp chambers, altered roof and floor dentin formation and a significant decrease in dentin/cementum formation by the age of 7 weeks. Histologically, the roof-forming odontoblasts were dramatically shorter and irregular in *Twist2-Cre*;*Xbp1* ^*CS/+*^ mice, compared to those in age-matched *Xbp1* ^*CS/+*^ control mice. Nevertheless, *in situ* hybridization and immunohistochemistry showed that increased XBP1S appeared to have no apparent effects on the expression of the odontoblast differentiation markers including *Dspp* and *Dmp1*. OSX is a transcription factor that is essential for osteoblast and odontoblast differentiation (Nakashima et al., 2002; Kim et al., 2015; Zhang et al., 2015; Yang et al., 2017). Moreover, it has been shown that XBP1S stimulates the expression of *Osx* during osteoblast differentiation (Tohmonda et al., 2011). However, immunohistochemistry demonstrated that the OSX immunostaining signals in *Twist2-Cre;Xbp1* ^*CS/+*^ mice were comparable to those in *Xbp1* ^*CS/+*^ mice (Supplementary Figure S2). Taken together, these findings suggest that constitutive expression of XBP1S negatively affected odontoblast function and dentin formation in mice. Further studies are needed to determine how sustained XBP1S caused a negative impact on odontoblast function in the future.

Accumulating evidence supports that the level and transcriptional activity of XBP1S is tightly regulated at the translational and post-translational levels during ER stress. First, XBP1S is only translated when the unspliced *XBP1U* mRNA is converted to the spliced mRNA by IRE1α RNase that is activated upon ER stress (Yoshida et al., 2001; Calfon et al., 2002). Second, XBP1S is subject to ubiquitin-mediated proteasomal degradation (Lee et al., 2018; Wang et al., 2019; Sun et al., 2020). Third, XBP1U also binds to XBP1S and enhances its degradation by proteasome (Tirosh et al., 2006; Yoshida et al., 2006). Lastly, it has been shown that other posttranslational modifications, such as phosphorylation, acetylation/deacetylation and sumoylation/desumoylation, can affect XBP1S protein stability and transcriptional activity (Lee et al., 2011; Wang et al., 2011; Jiang et al., 2012; Liu et al., 2016; Wang et al., 2016). Overall, these mechanisms ensure that XBP1S is only produced and functional when it is needed during ER stress, and that it is degraded once ER stress is relieved.

In summary, we have shown that persistent production of XBP1S adversely affected odontoblast function and dentin formation. These findings further highlight the importance of controlling the level and transcriptional activity of XBP1S within a cell. Loss of function study is warranted to determine if XBP1S is essential for odontoblast differentiation and function in the future.

## Data Availability

The original contributions presented in the study are included in the article/Supplementary Material, further inquiries can be directed to the corresponding author.
