# Supplementary material for: Constitutive expression of spliced X-box binding protein 1 inhibits dentin formation in mice
Source: Front Physiol. 2024 Jan 10;14:1319954. doi: 10.3389/fphys.2023.1319954 (PMC10809399; doi:10.3389/fphys.2023.1319954)
Supplement: Supplementary file 9 [file DataSheet1.pdf]

Supplementary Material

1 Supplementary Data

1.1 Immunohistochemistry

Immunohistochemistry (IHC) was performed to detect osterix (OSX) in the mandibular first molars of 3-week-old *Xbp1*$^{CS/+}$ and *Twist2-Cre;Xbp1*$^{CS/+}$ mice with a rabbit polyclonal anti-OSX antibody (1:1000, Abcam, Cambridge, MA). The detailed procedure is described in the main manuscript.

2 Supplementary Figures

Supplementary Figure S1: H&E staining of the mandibular first molars of 7-week-old mice. Shown are the images of H&E staining of a sagittally-sectioned mandibular first molars of 7-week-old *Xbp1*$^{CS/+}$ and *Twist2-Cre;Xbp1*$^{CS/+}$ mice. Note the reduced cementum (c) formed in *Twist2-Cre;Xbp1*$^{CS/+}$ mice, compared to that in *Xbp1*$^{CS/+}$ mice. rd, root dentin; c, cementum. Scale bars: 200 µm.

Supplementary Figure S2: Immunohistochemical staining of osterix (OSX). Shown are the representative images of IHC staining of OSX (signal in brown) in the mandibular first molars of 3-week-old *Xbp1*$^{CS/+}$ and *Twist2-Cre;Xbp1*$^{CS/+}$ mice (A). Each image in A is from the middle region of the crown of a sagittally-sectioned mandibular first molar. A1 and A2 are the higher magnification views of the roof-forming odontoblasts (box1) and floor-forming odontoblasts (box 2) in each image in A, respectively. rd, roof dentin; fd, floor dentin; rod, roof-forming odontoblasts; fod, floor-forming odontoblasts. Note that the OSX immunostaining signals in *Twist2-Cre;Xbp1*$^{CS/+}$ mice were comparable to those in *Xbp1*$^{CS/+}$ mice. Scale bars: 50 µm in A; 20 µm in A1-A2.

Supplementary Figure S3-10: The original images for the Western-blotting results shown in Figure 1C and 1D are provided as supplementary information. Please note that the last lane in each original image is loaded with the total cell lysate from the cells transiently transfected with a total of 3 µg of the *Xbp1* or *Xbp1s* minigene construct along with a construct expressing an IRE1α variant, K907A. The IRE1α K907A variant has the substitution mutation in the RNase domain of IRE1α, and shows compromised RNase activity in catalyzing the unconventional splicing of *XBP1U* mRNA into *XBP1S* mRNA. The results from IRE1α K907A are not presented in the main manuscript as they do not provide any further information.

Supplementary Figure S3: The original image for Figure 1C 1st panel XBP1S and XBP1U

Supplementary Figure S4: The original image for Figure 1C 2nd panel pIRE1α

Supplementary Figure S5: The original image for Figure 1C 3rd panel IRE1α

Supplementary Figure S6: The original image for Figure 1C 4th panel β-actin

Supplementary Figure S7: The original image for Figure 1D 1st panel XBP1S and XBP1U

Supplementary Figure S8: The original image for Figure 1D 2nd panel pIRE1α

Supplementary Figure S9: The original image for Figure 1D 3rd panel IRE1α

Supplementary Figure S10: The original image for Figure 1D 4th panel β-actin
